# Supplementary material for: Cross-protein transfer learning substantially improves disease variant prediction
Source: Genome Biol. 2023 Aug 7;24:182. doi: 10.1186/s13059-023-03024-6 (PMC10408151; doi:10.1186/s13059-023-03024-6)
Supplement: Supplementary file 1 — Additional file 1: Supplementary Information. This file contains all supplemental figures and tables along with their descriptions. [file 13059_2023_3024_MOESM1_ESM.pdf]

Additional File 1

Cross-protein transfer learning substantially improves disease variant prediction

Milind Jagota[1,*], Chengzhong Ye[2,*], Carlos Albors[1], Ruchir Rastogi[1],

Antoine Koehl[2], Nilah Ioannidis[1,3,4], and Yun S. Song[1,2,4,†]

[1]Computer Science Division, University of California, Berkeley, CA 94720
[2]Department of Statistics, University of California, Berkeley, CA 94720
[3]Chan Zuckerberg Biohub, San Francisco, CA 94158
[4]Center for Computational Biology, University of California, Berkeley, CA 94720

This file contains

- Supplementary Figures S1-S7
- Supplementary Tables S1-S4

[*]These authors contributed equally to this work.
[†]To whom correspondence should be addressed: yss@berkeley.edu

Supplementary Figures

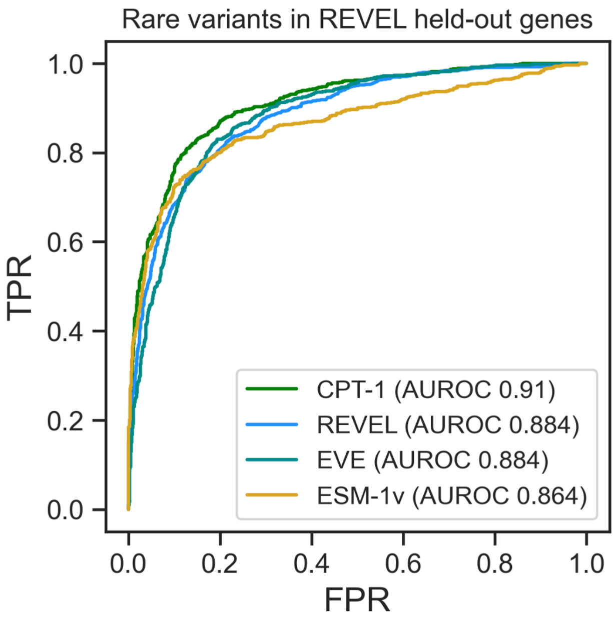

Figure S1: **Comparison with REVEL on rare variants.** CPT-1 outperforms REVEL on genes not seen by REVEL at train time (Figure 2). If we additionally restrict to the 50% of variants with lowest allele frequency (a cutoff of 3.7×10^{-4} in gnomAD), the margin over REVEL becomes larger. Also, REVEL does not outperform EVE in this rare variant subset.

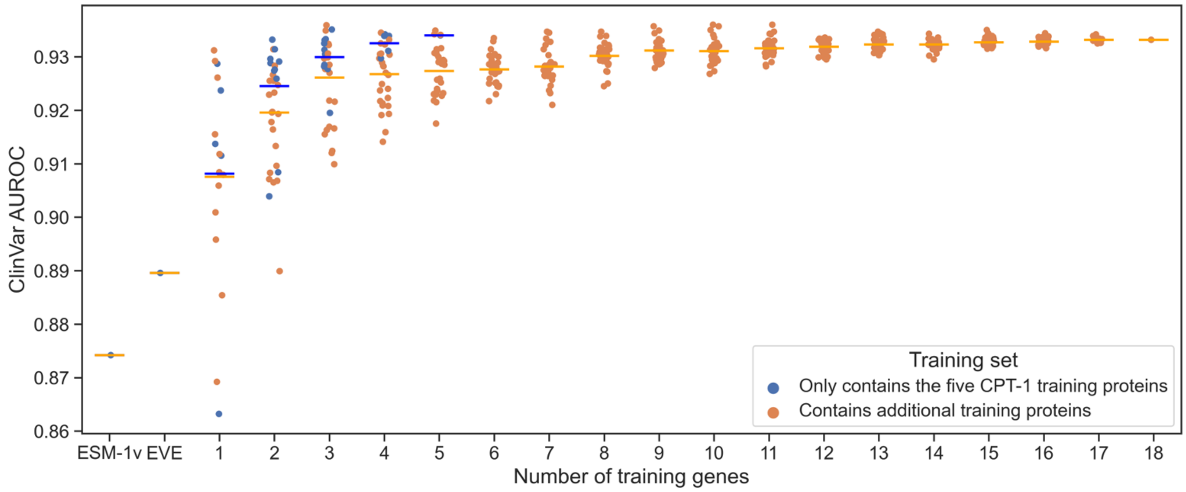

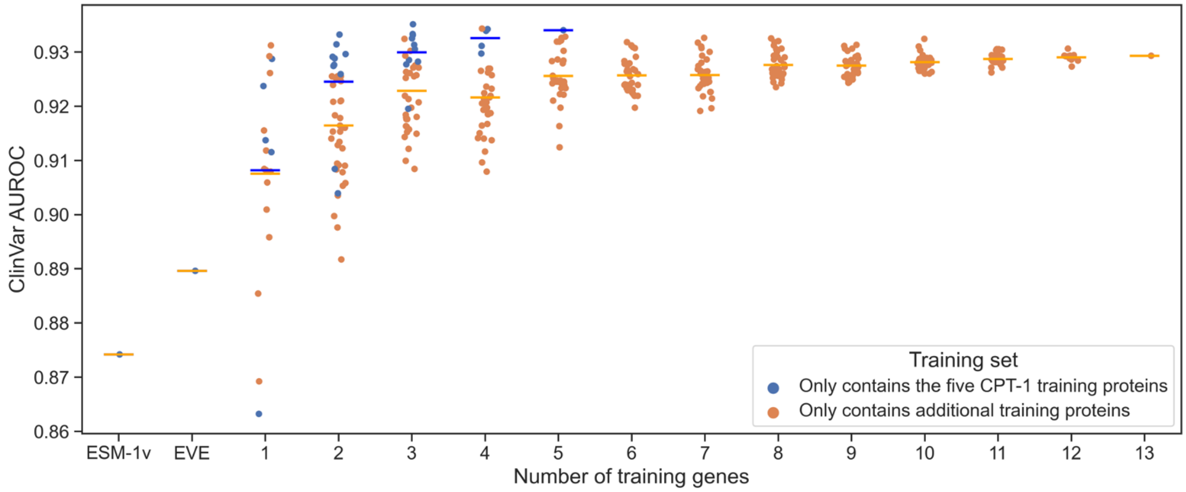

Figure S2: **Impact of choice of training genes.** CPT-1 is trained on functional assay data from five proteins generated by the same lab. We also examined the possibility of training on additional datasets from ProteinGym. Each dot indicates a specific choice of training genes, with the mean shown as a horizontal bar. (Top) Selecting from only the five main datasets yields better performance compared to a random, equal number from the full collection of 18 datasets. In addition, using all datasets does not outperform only using the five main datasets. (Bottom) Selecting from only the five main datasets yields better performance compared to a random, equal number from the 13 proteins from ProteinGym. These results demonstrate the value of using DMS data from the same assay and lab.

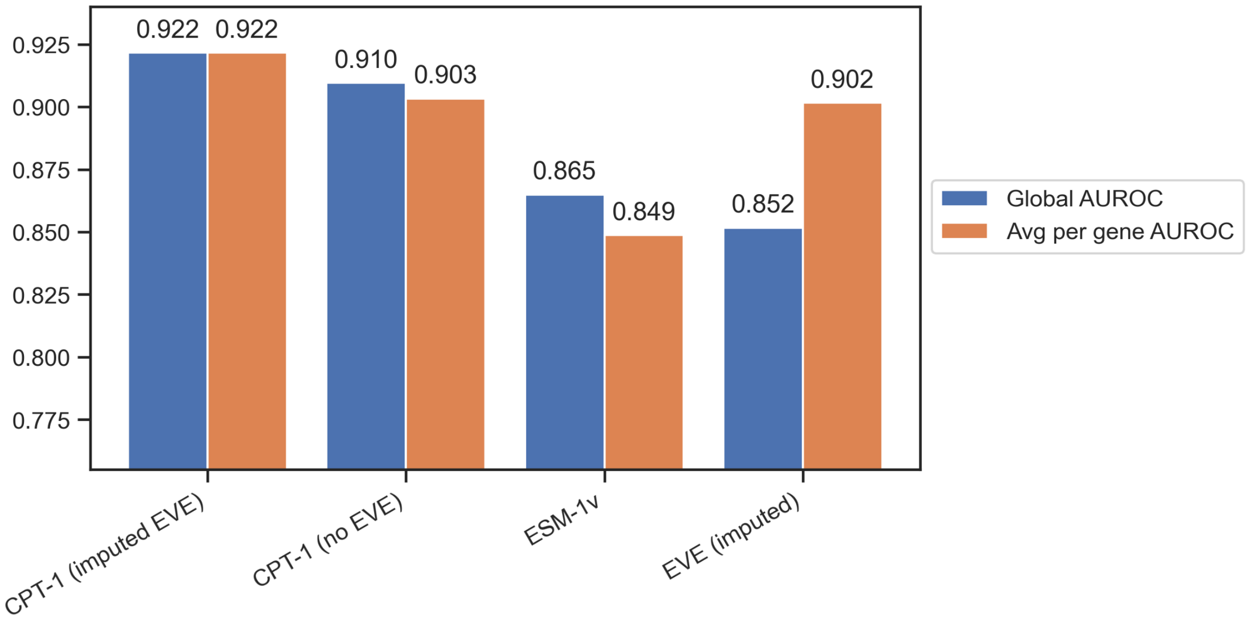

Figure S3: **AUROC results for positions where within-gene imputation of EVE predictions was performed.** EVE declines to make variant effect predictions at positions with low alignment quality in the EVE MSA. We imputed EVE predictions at these positions using a nearest-neighbors approach within the gene. Imputed predictions increase CPT-1 performance both in terms of overall AUROC and per-gene AUROC. ESM-1v performs worse on these low-alignment quality variants as well, compared to ESM-1v performance on other variants.

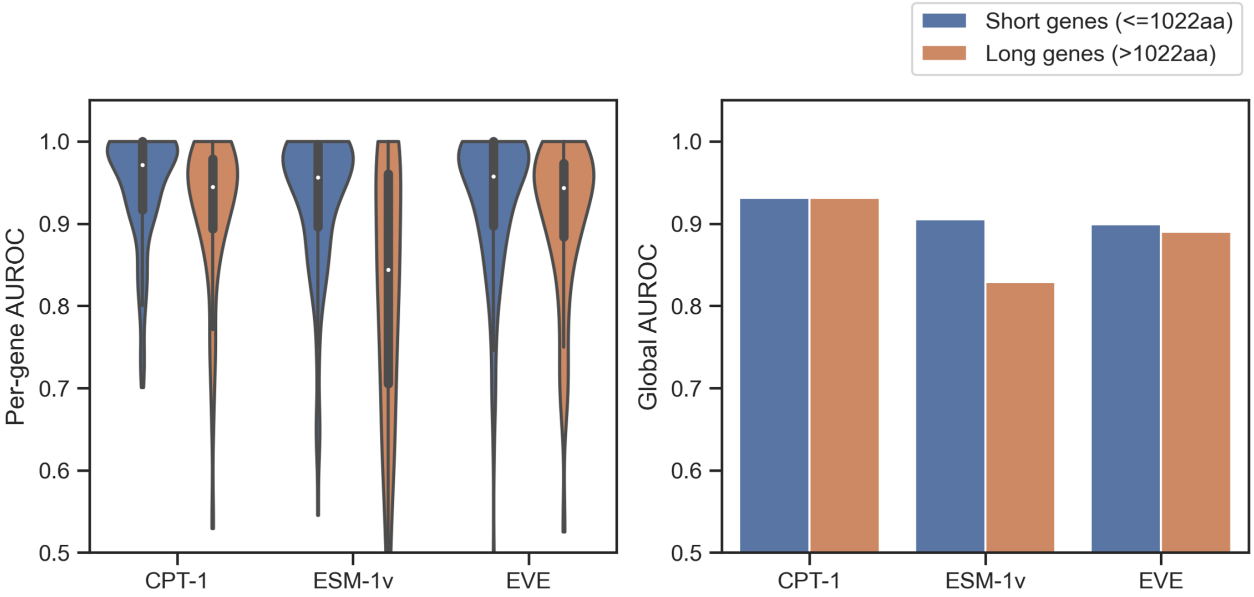

Figure S4: **Extending ESM-1v to long genes**. ESM-1v by default does not accept proteins longer than 1022 amino acids. To enable full coverage of human proteins, we developed a scheme to apply ESM-1v to longer proteins. ESM-1v with this extension performs worse on long genes, but CPT-1 does not suffer a performance drop. EVE performs as well on long proteins as on short.

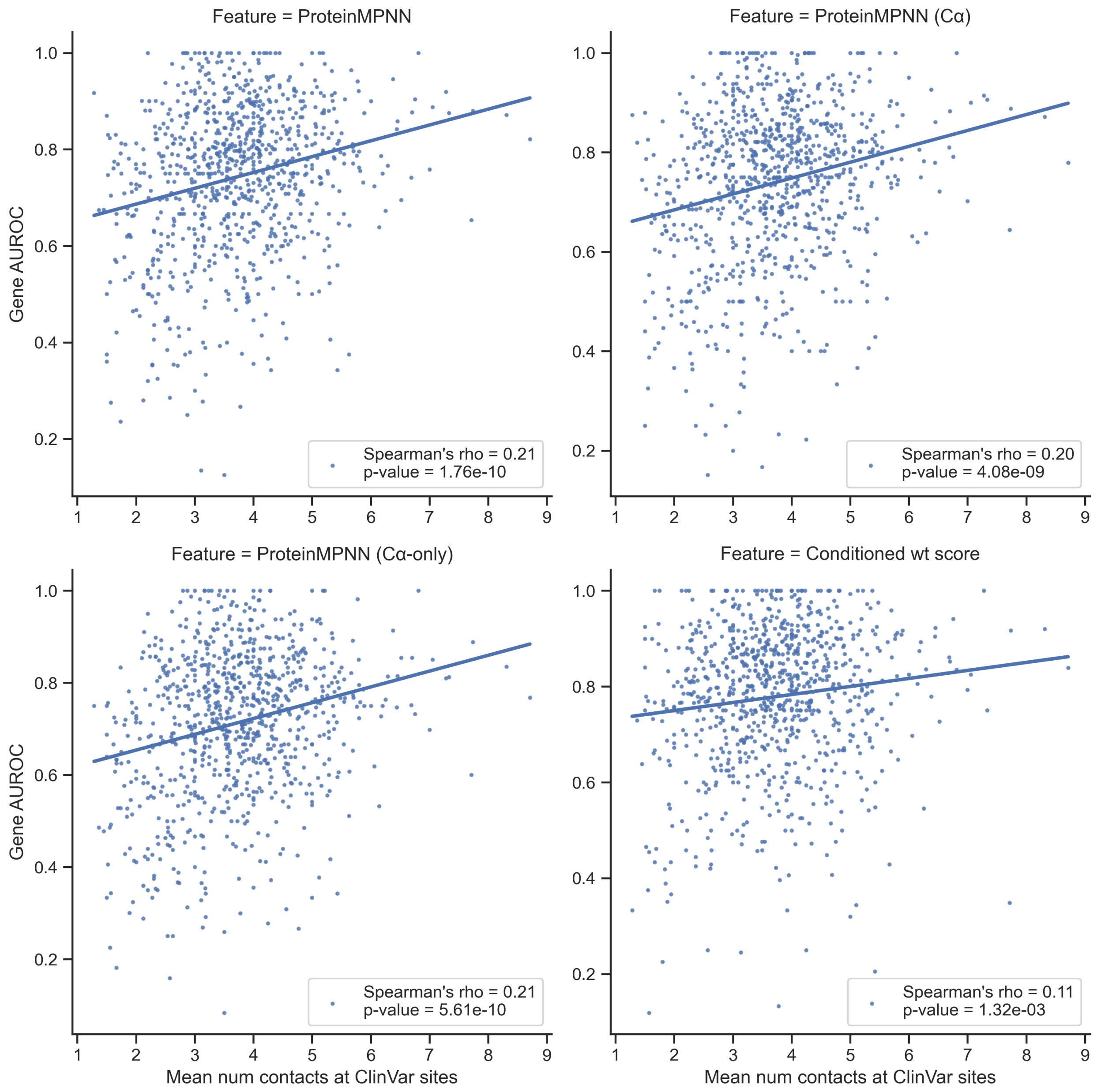

Figure S5: **Contact counts**. We calculated the number of sidechain-sidechain contacts formed by the position of each ClinVar variant and took the average of these counts in each protein. Structural features perform better in genes where ClinVar variant positions have more contacts. Contact count is itself a modest predictor of variant pathogenicity (0.69 AUROC), indicating that ClinVar variants in the core of proteins are more likely to be pathogenic.

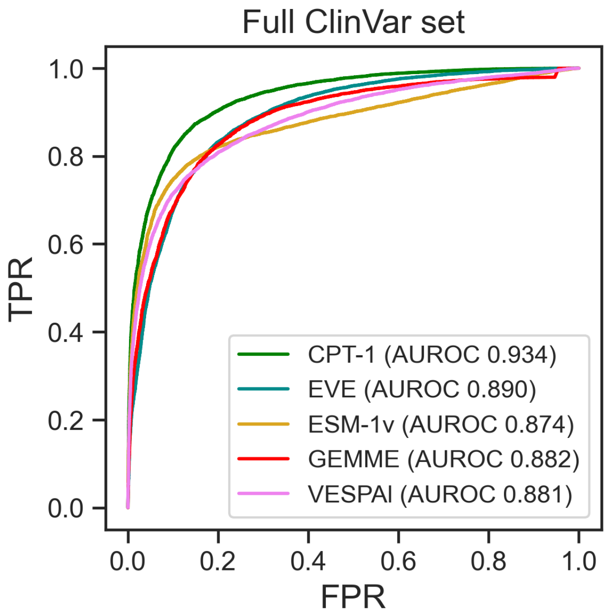

Figure S6: **Alternatives to EVE**. A limitation of CPT-1 is the reliance on EVE, which is the most computationally intensive feature to compute. We assessed the performance of alternative sequence variation methods GEMME [1] and VESPAl [2], which are faster to compute. These methods perform nearly as well as EVE, and could be substituted to more readily scale our method to other proteomes.

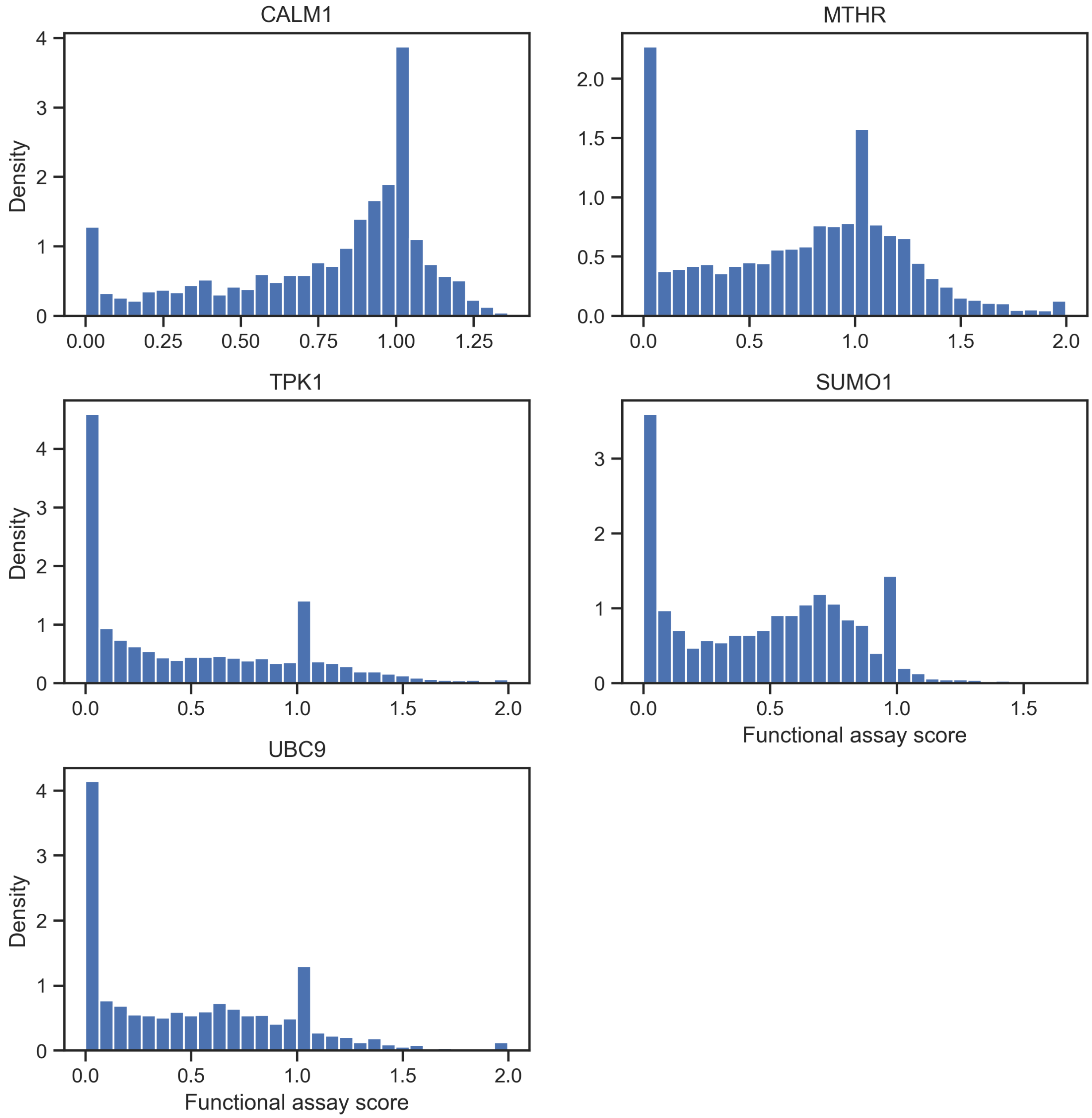

Figure S7: **Distribution of functional assay scores**. Functional assay scores for our five training proteins were generated by the same group with a standardized scale. A score of 0 is set to match the average effect of a nonsense (stop-gain) variant, while a score of 1 is set to match the average effect of a synonymous variant. Some datasets were truncated at 0, so we truncated all 5 at 0. Despite the relative homogeneity between these datasets, the distribution of variant scores differs significantly between genes, which led us to use binarization for our disease variant model.

Supplementary Tables

Feature	Type	Description	Source
EVE evolutionary index	General sequence variation	Scores from neural model of protein sequences in MSA	Frazer *et al.*, 2021 [3]
ESM-1v	General sequence variation	Scores from language model of protein sequences	Meier *et al.*, 2021 [4]
100 vertebrate mut. freq.	Vertebrate alignment	Mutant frequency in orthologous proteins from 100 vertebrates	UCSC Genome Browser [5, 6]
30 mammal mut. freq.	Vertebrate alignment	Mutant frequency in orthologous proteins from 30 mammals	UCSC Genome Browser [5, 7]
Conditioned wildtype score	Structure + Sequence variation	Wildtype freq. in EVE MSA, conditioned on contact residues matching human protein	Novel [8, 9]
Vanilla ProteinMPNN	Structure	Scores from neural network on protein structure and partial sequence	Dauparas *et al.*, 2022 [10]
BLOSUM Index 1	Amino Acid Descriptor	The first component of BLOSUM62-derived indices.	Georgiev, 2009 [11]
ST6	Amino Acid Descriptor	The sixth principal component of ST-scales.	Yang *et al.*, 2010 [12]
VHSE6	Amino Acid Descriptor	The sixth principal component (electrical properties) of VHSE-scales.	Mei *et al.*, 2005 [13]

Table S1: **Final selected features for CPT-1**. CPT-1 is trained on features based on general sequence variation, sequence variation in vertebrates, protein structure, and amino acid descriptors. Certain features are imputed across genes for the whole-proteome scale version of CPT-1 (underlined). For all amino acid descriptor features, the difference from wild-type to mutant amino acid is used. All computed features are released on Zenodo [8, 9].

Model	Overall AUROC	Avg gene AUROC	Median gene AUROC	Spec @ 95% Sens	Sens @ 95% Spec
CPT-1	0.934	0.933	0.958	0.675	0.698
CPT-1 (no vtMSA)	0.925	0.925	0.951	0.607	0.693
CPT-1 (no structure)	0.93	0.932	0.958	0.650	0.696
CPT-1 with imputed EVE	0.923	0.918	0.940	0.611	0.662
EVE	0.89	0.919	0.949	0.548	0.512
ESM-1v	0.874	0.877	0.931	0.273	0.640
100vt wt frequency	0.865	0.865	0.890	0.508	0.00
Vanilla ProteinMPNN	0.781	0.765	0.791	0.134	0.390
Conditioned mt score	0.826	0.844	0.881	0.287	0.435
Conditioned wt score	0.719	0.807	0.819	0.196	0.185

Table S2: **Detailed classification performance on ClinVar missense variants.** CPT-1 outperforms baselines on all metrics. Per-gene ClinVar AUROC is calculated in 274 genes with at least four benign and four pathogenic ClinVar variants. The margin over EVE and ESM-1v depends heavily on vertebrate alignments and, to a lesser extent, on structure. The cross-gene imputation version of CPT-1 is used to produce predictions at whole-proteome scale; it performs worse than using true feature values, but outperforms EVE and ESM-1v. 100-vertebrate MSA wild-type frequency is a powerful baseline in the high-sensitivity regime. Structural features perform worse alone but have enough non-redundant signal to be worth including.

Protein	CPT-1	EVE	ESM-1v
Average	**0.448**	0.380	0.412
SUMO1	**0.640**	0.606	0.580
UBC9	**0.584**	0.574	0.558
TPK1	0.374	0.314	**0.396**
MTHR	**0.330**	0.293	0.314
CALM1	0.301	0.291	**0.318**
SC6A4	**0.567**	0.520	0.542
YAP1	0.560	**0.617**	0.441
P53	**0.555**	0.452	0.553
BRCA1	**0.555**	0.515	0.450
VKOR1	**0.534**	0.464	0.474
PTEN	**0.507**	0.360	0.454
RASH	0.467	**0.480**	0.405
A4	**0.456**	0.343	0.435
TPOR	**0.439**	0.352	0.400
MSH2	**0.417**	0.394	0.400
KCNH2	**0.358**	0.023	0.295
SYUA	**0.304**	0.156	0.267
SCN5A	0.120	0.082	**0.135**

Table S3: **Detailed regression performance on DMS datasets of human proteins.** Spearman's ρ of CPT-1, EVE, and ESM-1v are shown for eighteen DMS datasets of human proteins. The top five are the CPT-1 training proteins and the remaining thirteen are from ProteinGym. The highest performing method for each protein is bolded.

Model	Overall AUROC	Avg gene AUROC
CPT-1 (30, 50 binarize)	0.930	0.928
CPT-1 (30, 60 binarize)	0.931	0.928
CPT-1 (30, 70 binarize)	0.930	0.927
CPT-1 (40, 50 binarize)	0.933	0.929
CPT-1 (40, 60 binarize)	0.934	0.934
CPT-1 (40, 70 binarize)	0.933	0.930
CPT-1 (50, 50 binarize)	0.933	0.930
CPT-1 (50, 60 binarize)	0.934	0.933
CPT-1 (50, 70 binarize)	0.933	0.931
ESM-1v	0.874	0.876
EVE	0.890	0.919

Table S4: **CPT-1 performance is stable under different choices of training data binarization.** We binarized training functional assay data for CPT-1 based on percentile within each protein; variants with score below the 40th percentile were labeled pathogenic, and variants with score above the 60th percentile were labeled benign. Changing these thresholds does not change performance on ClinVar variants much, and CPT-1 always outperforms baselines.

References

[1] Elodie Laine, Yasaman Karami, and Alessandra Carbone. Gemme: a simple and fast global epistatic model predicting mutational effects. *Molecular Biology and Evolution*, 36(11):2604–2619, 2019.

[2] Céline Marquet, Michael Heinzinger, Tobias Olenyi, Christian Dallago, Kyra Erckert, Michael Bernhofer, Dmitrii Nechaev, and Burkhard Rost. Embeddings from protein language models predict conservation and variant effects. *Human Genetics*, 141(10):1629–1647, 2022.

[3] Jonathan Frazer, Pascal Notin, Mafalda Dias, Aidan Gomez, Joseph K Min, Kelly Brock, Yarin Gal, and Debora S Marks. Disease variant prediction with deep generative models of evolutionary data. *Nature*, 599(7883):91–95, 2021.

[4] Joshua Meier, Roshan Rao, Robert Verkuil, Jason Liu, Tom Sercu, and Alex Rives. Language models enable zero-shot prediction of the effects of mutations on protein function. *Advances in Neural Information Processing Systems*, 34:29287–29303, 2021.

[5] W James Kent, Charles W Sugnet, Terrence S Furey, Krishna M Roskin, Tom H Pringle, Alan M Zahler, and David Haussler. The human genome browser at UCSC. *Genome Research*, 12(6):996–1006, 2002.

[6] Mathieu Blanchette, W James Kent, Cathy Riemer, Laura Elnitski, Arian FA Smit, Krishna M Roskin, Robert Baertsch, Kate Rosenbloom, Hiram Clawson, Eric D Green, et al. Aligning multiple genomic sequences with the threaded blockset aligner. *Genome Research*, 14(4):708–715, 2004.

[7] Kerstin Lindblad-Toh, Manuel Garber, Or Zuk, Michael F Lin, Brian J Parker, Stefan Washietl, Pouya Kheradpour, Jason Ernst, Gregory Jordan, Evan Mauceli, et al. A high-resolution map of human evolutionary constraint using 29 mammals. *Nature*, 478(7370):476–482, 2011.

[8] Chengzhong Ye, Milind Jagota, Carlos Albors, Ruchir Rastogi, Antoine Koehl, Nilah Ioannidis, and Yun S. Song. CPT-1 whole-proteome feature matrices (EVE set). Zenodo. https://doi.org/10.5281/zenodo.8137051 (2023).

[9] Chengzhong Ye, Milind Jagota, Carlos Albors, Ruchir Rastogi, Antoine Koehl, Nilah Ioannidis, and Yun S. Song. CPT-1 whole-proteome feature matrices (no-EVE set). Zenodo. https://doi.org/10.5281/zenodo.8137108 (2023).

[10] Justas Dauparas, Ivan Anishchenko, Nathaniel Bennett, Hua Bai, Robert J Ragotte, Lukas F Milles, Basile IM Wicky, Alexis Courbet, Rob J de Haas, Neville Bethel, et al. Robust deep learning–based protein sequence design using ProteinMPNN. *Science*, page eadd2187, 2022.

[11] Alexander G Georgiev. Interpretable numerical descriptors of amino acid space. *Journal of Computational Biology*, 16(5):703–723, 2009.

[12] Li Yang, Mao Shu, Kaiwang Ma, Hu Mei, Yongjun Jiang, and Zhiliang Li. ST-scale as a novel amino acid descriptor and its application in QSAM of peptides and analogues. *Amino Acids*, 38(3):805–816, 2010.

[13] HU Mei, Zhi H Liao, Yuan Zhou, and Shengshi Z Li. A new set of amino acid descriptors and its application in peptide QSARs. *Peptide Science: Original Research on Biomolecules*, 80(6):775–786, 2005.
