# Supplementary material for: Cross-protein transfer learning substantially improves disease variant prediction
Source: Genome Biol. 2023 Aug 7;24:182. doi: 10.1186/s13059-023-03024-6 (PMC10408151; doi:10.1186/s13059-023-03024-6)
Supplement: Supplementary file 2 — Additional file 2. Review History. [file 13059_2023_3024_MOESM2_ESM.docx]

Review History

**First round of review**

**Reviewer 1**

**Were you able to assess all statistics in the manuscript, including the appropriateness of statistical tests used?**

Yes, and I have assessed the statistics in my report.

**Comments to author:**

The manuscript entitled « Cross-protein transfer learning substantially improves zero-shot prediction of disease variant effects » by Jagota et al. reports on a machine learning method for predicting the pathogenicity of mutations. The method, CPT-1, includes a variety of sequence-based and structure-based descriptors and leverages the power of recently developed deep learning methods and protein language models. The authors find that considering amino acid frequencies in alignments of vertebrate and mammal sequences contributes to improving the predictions and sometimes is even more informative than much more sophisticated models. This finding is interesting and opens new directions. They also find that the contribution of 3D structure is small.

However, the improvement of CPT-1 over ESM-1v and EVE is modest. Moreover, the dependence on EVE makes it difficult for the method to scale to entire proteomes. Have the authors considered other options, such as GEMME (http://www.lcqb.upmc.fr/GEMME/Home.html), which performs very well (see: https://www.proteingym.org/substitutions) and requires much less computing time? By the way, there are also alternatives to ESM-1b (see VESPA, which is based on ProtT5 on https://www.proteingym.org/substitutions).

- Although they learn from different inputs (MSAs versus single sequences), EVE and ESM-1b scores are redundant to some extent. Did the authors consider using only one or the other? Or of using the scores of EVE before the Gaussian fitting (which is some extra layer to go from DMS predictions to clinical significance predictions, it seems somewhat redundant to the regression the authors are applying).

- What would be the gain brought by CPT-1 over few-shot predictions using ESM-1b? Could the authors comment on the potential of cross-protein transfer with language models?

- Highlighting a few proteins to emphasise the importance of this or that feature in some contexts may be a bit misleading since the set of annotated variants with known effect is very small. So some trend may come for a bias in the sampling of the mutations. Could the authors add some discussion about this point?

- In several places, the clarity of the explanations could be improved. Maybe adding some equations in the Methods section would help. I did not understand for instance the part about the two most conserved sidechain-sidechain contacts for each position. Where does the mutant come into play? Why 2 ? Why not take the top pairs for instance?

- Is there a code associated with the work? I did not see any link to a GitHub repo or else with some functioning code and/or the data necessary to reproduce the reported results.

- is the binarisation of the DMS into top 40% and lowest 40% realistic? Do we expect to have balanced labels for pathogenicity and neutrality ? My intuition would be that there are much fewer pathogenic mutations than neutral ones.

- « Spec@«  is very unclear on Fig. 2B. I suggest to remove it and put « specificity » as the label of the y-axis.
- As can be seen on Fig. 2D, the performance vary greatly from one protein to another. In particular the poor performance achieved for CALM1 and MTHR questions the pertinence of training on these proteins. Could the author comment a bit on this issue?

**Reviewer 2**

**Were you able to assess all statistics in the manuscript, including the appropriateness of statistical tests used?**

There are no statistics in the manuscript.

**Comments to author:**

Jagota et al. present a new model for predicting variant effects, CPT-1. The main interesting features of the model are that it incorporates a vertebrate alignment feature and that it is trained on deep mutational scanning (DMS) datasets. On the plus side, CPT-1 outperforms two other models, EVE and ESM-1v. On the minus side, I'm not convinced the DMS data is actually adding that much. Relatedly, the authors used a very small fraction of the available DMS data, and didn't really motivate or justify this choice. I'm also not sure there is much new here, especially if vertebrate alignments have been incorporated into predictors before. Overall the work is technically good, though I have some concerns, detailed below, about how things are described.

General comments

The main claim of the paper is that DMS data are useful in training human disease variant predictors. However, I don't feel that the paper really proves this claim. The authors make a nice model with a solid set of features (most notably the vertebrate alignments). Then, they train it using DMS data. However, I wonder what would happen if they merely used the features in an unsupervised way, perhaps analogous to EVE, to predict human disease variants. Here, they could compute some individual score summarizing all the features and then examine how human disease variants distributed relative to such a score. One way to do this would be to just use a naive logistic model where the feature weights were uniform. The feature-by-feature analysis they present suggests that they might do excellently. Put another way, do the DMS data do any real work here, or are they just a useful, but complicated, way to partition the feature space?
The authors pick 5 DMS datasets to train in. But, there are hundreds of available DMS datasets, many of which are focused on human disease-relevant. Some of these datasets outperform the ones the authors chose. They should better justify their choice of these five - just stating that they wanted to have more uniform data is insufficient. They should also explore what would happen if more datasets were included. They suggest that having datasets from a single lab/assay is important. Is that really so? Or, can lab-to-lab and assay-to-assay bias be dealt with?
The CPT model enters a crowded space of predictors seeking to inform human disease variant interpretation. The authors should do a better job in the introduction and discussion of contextualizing CPT in this crowded space. Is CPT really better than, say, REVEL, which many clinicians use? If so, why? If not, why is it useful?
There have been a variety of previous efforts to use functional assay data to train predictors. The current work leverages more sophisticated ML methods and improved features, but the authors should cite and at least briefly discuss how their work relates to the previous work.
The authors use the classifications "benign or deleterious." These may not be the best terms. In general, clinicians classify variants into one of five categories (benign, likely benign, VUS, likely pathogenic, or pathogenic). The title suggests that CPT predicts "disease variant effects." So, is it a pathogenicity predictor? If so, shouldn't the classes be benign and pathogenic? Or, if it is predicting variant effects rather than variant pathogenicity, which is what training on DMS data would suggest, then maybe terms like "functionally normal and functionally abnormal" would be better? Since one the authors led the development of the REVEL pathogenicity predictor, I think the authors can do a better job of clarifying whether CPT is a pathogenicity predictor and using appropriate terms.
They use Clinvar variants to validate the model. Clinvar is notoriously full of old and low-quality interpretations, which could actually make CPT appear worse than it is. It would be interesting to see how CPT performs on variants from at least 1 star submitters in the last 5-10 years.
The authors could do a much better job of labeling plot axes in figures. Many plots have labels like "Fraction" or "Spearman's rho" or "AUROC," but in different figures different fractions of things are being compared (e.g. DMS data or Clinvar data, etc). Improving the specificity of the labels would help reader comprehension.
The authors make a version of CPT that uses imputed features. This is what enables them to go from the relatively small fraction of human proteins for which EVE predictions are available to 90% of human proteins. However, they don't present the performance of the model using these imputed features in the main text, describing results only vaguely and pointing to the supplement. I think the authors should put a version of supplementary figure 6 in the main text and substantially expand the section of the paper dealing with the imputation. They should fully and fairly describe the effects of using imputed features. They should say how many genes have EVE-derived features and how many have imputed features. I didn't check, but they should also note whether EVE-derived or imputed features were used for each prediction available in the GitHub repository.
The authors should upload their predictions to Zenodo or some other external, versioned repository that does not allow data deletion. That way, CPT predictions will be available outside the author's GitHub repository.

Specific comments

Page 2 - The authors write "Functional assays via deep mutational scanning (DMS) experiments have been used to measure the effects of missense variants in non-human systems at higher throughput." This is confusing because many of the DMS experiments that have been done have been in cultured human cell lines. Clarifying or omitting "non-human systems" seems advisable.

Page 2 - The authors write " However, the effects of missense mutations often involve aspects of protein function that are specific to humans or species similar to humans." This statement should have a citation. Moreover, I'm not sure I agree. And, the logic of the paragraph, which starts by asserting (correctly) that EVE and ESM-1v draw information from large MSAs composed mostly of non-human sequences and also that they perform quite well at predicting the effect of human variants on disease, contradicts the quoted paragraph. The authors shoudl clarify their argument.

Page 2 - The authors state "...but such models can be inaccurate due to unknown biases in the collection of these data." This is a vague statement that should be clarified and cited.

Page 3/Figure 2 - The authors show that CPT improves specificity at high sensitivity, which is great. It might help the reader appreciate how important these gains are if the authors used real numbers of variants as opposed to fractions/proportions.

Page 4 - The performance of the approach on predicting held-out DMS datasets is quite low.and not really better than previous approaches. Why? What is the relationship between being able to predict DMS data (moderate) and clinical variants (pretty good)? A discussion of these matters would be interesting.

Page 4 - The authors again make the argument that variants have human-specific effects that aren't well represented by MSAs drawn from diverse organisms. Again, I think this assertion needs more justification/citation/explanation.

Page 4 and elsewhere - The authors use the phrase "local-timescale alignment." I think they mean an alignment of closely related vertebrates and mammals. If so, then I suggest they ditch or introduce the jargon which many will not understand. If they mean something else, they should explain.

Page 4 - The authors assert "These traits mean that conservation in these alignments is likely to be non-redundant with EVE and ESM-1v while being more specific to function and organism." They should examine the redundancy of conservation within their alignments as compared to those used for EVE/ESM-1v.

Page 4 - The authors state that others have previously shown that local-timescale alignments are powerful predictors of pathogenicity. Then, they go on to analyze how their own local timescale alignment is a powerful predictor of pathogenicity. It would be helpful for the reader to know what is new here, or if the authors are just reproducing what others have done/shown.

Page 4- "Furthermore, if a mutant allele occurs at all in either the 100-vertebrate or 30-mammal MSA, it can be inferred to be benign with high probability (Figure 3B)" The authors could and should be more quantitative here.

Pages ⅘ - The authors note that their version of CPT with no vtMSA performs substantially worse on clinical data. It's interesting that it really craters at high sensitivity. Why is that? The authors should explore/explain.

Page 5/Figure 3D - The authors highlight two genes where vtMSA is particularly important. Related to the previous comments, I feel this analysis could be improved significantly. Why were these genes chosen? What makes vtMSA useful for a gene (e.g. is it having a deeper MSA/gene length? Something else?)? Additionally, what variants does the vtMSA feature tend to get wrong?

Page 5 - The authors mention "a hand-designed feature that combines a known structure with conservation in the EVE MSA (Methods)." They should say more about it in the main text.

Page 6 - Many readers of a generalist journal like GB will be unfamiliar with model perplexity. It should be introduced.

Page 6 - The authors "imputed all features that depend on the EVE MSA across genes." They should present an analysis of the quality of these imputed features.

Page 6 - The authors state "We assessed this version of CPT-1 in the EVE dataset of preprocessed ClinVar variants and found that the model still outperformed EVE and ESM-1v." Here, the authors make vague claims about the performance of the expanded CPT model, referring readers to the supplementary figure. Performance data should be presented in main figures.

Page 6/supplementary figure 6 - The avg. per-gene AUROC for EVE predictions on "true" EVE genes is substantially better than the global AUROC. This seems weird to me, and should be explained.

**Authors Response**

**Point-by-point responses to the reviewers’ comments:**

**Point-by-point Response to Reviews**

GBIO-D-22-01780

We thank the reviewers for their thoughtful comments, which helped to improve our manuscript. We have carried out extensive further analyses and thoroughly revised the manuscript to address the reviews. In particular, we have explored the possibility of training on a larger collection (eighteen) of DMS datasets pulled from ProteinGym. The revised manuscript describes the impact of the choice of DMS datasets and the training scheme on the model performance. The rationale behind our model design is now better explained and we demonstrate more clearly that training on DMS data indeed helps to improve disease variant pathogenicity prediction. In addition, following Reviewer 2’s suggestion, we have added a benchmarking study against REVEL, a supervised method widely used by clinicians; we show that CPT-1 compares favorably with REVEL.

The editor raised a concern regarding “the lack of technological novelty.” Our model employs state-of-the-art unsupervised sequence models as well as AlphaFold2 protein structure predictions and ProteinMPNN. We strongly believe that utilizing these recent advances in machine learning is innovative and investigating their potential to improve variant effect prediction, as we have done in our work, will help to advance the field. We also note that utilizing these models to make comprehensive variant effect predictions (at every site and to all 19 alternate amino acids) for 90% of human proteins requires a substantial amount of work, which we have carried out; we believe that the model predictions released with our publication will make a valuable resource for the community.

Our point-by-point response to reviews is provided below.

**Reviewer #1:** The manuscript entitled « Cross-protein transfer learning substantially improves zero-shot prediction of disease variant effects » by Jagota et al. reports on a machine learning method for predicting the pathogenicity of mutations. The method, CPT-1, includes a variety of sequence-based and structure-based descriptors and leverages the power of recently developed deep learning methods and protein language models. The authors find that considering amino acid frequencies in alignments of vertebrate and mammal sequences contributes to improving the predictions and sometimes is even more informative than much more sophisticated models. This finding is interesting and opens new directions. They also find that the contribution of 3D structure is small.

However, the improvement of CPT-1 over ESM-1v and EVE is modest.

High sensitivity is crucial for clinical applications of variant effect prediction. In this regime, our model has substantially higher specificity compared to ESM-1v and EVE. For example, at 95% sensitivity, the specificity of our model is 68%, while ESM-1v and EVE achieve only 27% and 55%, respectively. We believe that this is no small feat. Reviewer 2 also recognizes this significance in their review:

1

*“The authors show that CPT improves specificity at high sensitivity, which is great. It might help the reader appreciate how important these gains are if the*

*authors used real numbers of variants as opposed to fractions/proportions.”*

Moreover, the dependence on EVE makes it difficult for the method to scale to entire proteomes. Have the authors considered other options, such as GEMME (<http://www.lcqb.upmc.fr/GEMME/Home.html),> which performs very well (see: <https://www.proteingym.org/substitutions)> and requires much less computing time? By the way, there are also alternatives to ESM-1b (see VESPA, which is based on ProtT5 on <https://www.proteingym.org/substitutions).>

Thank you for the suggestion. We evaluated the accuracy of GEMME and VESPA and found that they perform comparably but slightly worse than EVE and ESM-1v (see plot below). These features therefore could be used to make our model faster to apply to other proteomes while only slightly reducing performance. For the purposes of this paper, which focuses on the human proteome, we have kept EVE and ESM-1v in our model.

- Although they learn from different inputs (MSAs versus single sequences), EVE and ESM-1b scores are redundant to some extent. Did the authors consider using only one or the other? Or of using the scores of EVE before the Gaussian fitting (which is some extra layer to go from DMS predictions to clinical significance predictions, it seems somewhat redundant to the regression the authors are applying).

We have replaced the EVE Gaussian mixture scores with the log probabilities, and this slightly improved the performance of CPT-1. However, we found that including both EVE and ESM-1v is useful. For example, the performance of CPT-1 without EVE is worse than the full model (Figure 6C-D, reproduced below). Also, averaging EVE and ESM-1v improves performance over either model alone (Figure 3A, reproduced below). We therefore have kept both EVE and ESM–1v in our model.

- What would be the gain brought by CPT-1 over few-shot predictions using ESM-1b? Could the authors comment on the potential of cross-protein transfer with language models?

The original ESM-1v paper explored fine-tuning ESM-1v in the context of deep mutational scanning prediction, observing only very minor increases (and in some cases decreases) in performance. We therefore expect that this strategy would not match the performance of CPT-1. A comprehensive, proteome-wide evaluation of this approach is beyond the scope of the current paper. Additionally, VESPA tested downstream learning on language model embeddings, but did not significantly improve on the performance of ESM.

- Highlighting a few proteins to emphasise the importance of this or that feature in some contexts may be a bit misleading since the set of annotated variants with known effect is very

3

small. So some trend may come for a bias in the sampling of the mutations. Could the authors add some discussion about this point?

We have removed the discussion of individual genes. Instead, we describe broad traits of genes where vertebrate alignments are useful, reproduced below.

“At a per-gene level, adding vertebrate alignments is neutral or beneficial to the performance of CPT-1 in 84% of genes. The genes where vertebrate alignments help the most (top 10%) are more challenging genes in general, with lower average AUROCs for CPT-1, ESM-1v, and EVE. In addition, they have more shallow MSAs (average depth of 6,600 compared to 10,000 in all genes) and are longer (68% are longer than 1000 amino acids, compared to 44% in all genes). These discrepancies suggest that vertebrate alignments may be more useful in more complex human genes, which are more recently evolved and harder to model through general sequence homology.”

- In several places, the clarity of the explanations could be improved. Maybe adding some equations in the Methods section would help. I did not understand for instance the part about the two most conserved sidechain-sidechain contacts for each position. Where does the mutant come into play? Why 2 ? Why not take the top pairs for instance?

We have significantly expanded the discussion of these particular features, in both the main text and the Methods. In the main text, we write:

“First, we included multiple versions of the deep neural network ProteinMPNN (which takes structure as input). Second, we included two hand-designed features that combine a known structure with conservation in the EVE MSA. For the latter features, we aimed to compute wild-type and mutant frequencies conditioned on the structural environment being the same as in the human protein. To achieve this, we first find for each position all other positions which are in contact in the AlphaFold structure. We then filter the EVE MSA to sequences where these positions have the same amino acids as in the human sequence. However, we perform this filtering using only a maximum of two contact residues, to ensure the number of sequences does not become too small. We define these features precisely in the Methods.”

In the Methods, we write:

“We also included two hand-designed structural features which we found perform well on functional assay data. These features aim to capture sequence variation in the EVE MSA conditioned on the structural environment around a position matching the environment in human proteins. Using the AlphaFold2 structure and the EVE MSA, we calculated for each position all other positions that form a sidechain-sidechain contact to it. Sidechain-sidechain contacts were calculated using the Probe software (see **Datasets**). Next, for each position, we filter the EVE MSA to sequences where the contact residues for that position have the same amino acids as in the human sequence. However, we only use the two contact residues where the human amino acid appears most frequently in the EVE MSA, to keep the number of sequences from

becoming too small. We additionally only allowed conditioning on residues with pLDDT greater than 70 in the AlphaFold2 structure, and residues with pLDDT less than 70 did not have any conditioning used. Finally, for each position, we compute the frequency of the human allele and all possible alternative amino acids in the filtered MSA. These features are the *conditioned wild-type score* and *conditioned mutant score*, respectively.”

- Is there a code associated with the work? I did not see any link to a GitHub repo or else with some functioning code and/or the data necessary to reproduce the reported results.

We have updated the GitHub repository (<https://github.com/songlab-cal/CPT)> with codes and data for reproducing the main results in the manuscript.

- is the binarisation of the DMS into top 40% and lowest 40% realistic? Do we expect to have balanced labels for pathogenicity and neutrality ? My intuition would be that there are much fewer pathogenic mutations than neutral ones.

We conducted a sweep of several different choices of binarization, including asymmetric binarization in both directions (Supplemental Table S4, reproduced below). We found that this had very little impact on the performance of CPT-1.

- « Spec@« is very unclear on Fig. 2B. I suggest to remove it and put « specificity » as the label of the y-axis.

Thank you for pointing this out. We have made this change, as shown below:

5

- As can be seen on Fig. 2D, the performance vary greatly from one protein to another. In particular the poor performance achieved for CALM1 and MTHR questions the pertinence of training on these proteins. Could the author comment a bit on this issue?

We experimented with all choices of which training proteins to use (Figure 3B, reproduced below). We found that removing CALM1 and MTHR did not improve model performance, indicating that these datasets contain useful signal to learn from despite being challenging to predict.

In addition, we have tested CPT-1 on a significantly expanded set (eighteen) of functional assay datasets which includes the original five (Supplemental Figure S2). We observe consistently improved performance over ESM-1v and EVE.

**Reviewer #2:** Jagota et al. present a new model for predicting variant effects, CPT-1. The main interesting features of the model are that it incorporates a vertebrate alignment feature and that it is trained on deep mutational scanning (DMS) datasets. On the plus side, CPT-1 outperforms

6

two other models, EVE and ESM-1v. On the minus side, I'm not convinced the DMS data is actually adding that much. Relatedly, the authors used a very small fraction of the available DMS data, and didn't really motivate or justify this choice. I'm also not sure there is much new here, especially if vertebrate alignments have been incorporated into predictors before. Overall the work is technically good, though I have some concerns, detailed below, about how things are described.

General comments

The main claim of the paper is that DMS data are useful in training human disease variant predictors. However, I don't feel that the paper really proves this claim. The authors make a nice model with a solid set of features (most notably the vertebrate alignments). Then, they train it using DMS data. However, I wonder what would happen if they merely used the features in an unsupervised way, perhaps analogous to EVE, to predict human disease variants. Here, they could compute some individual score summarizing all the features and then examine how human disease variants distributed relative to such a score. One way to do this would be to just use a naive logistic model where the feature weights were uniform. The feature-by-feature analysis they present suggests that they might do excellently. Put another way, do the DMS data do any real work here, or are they just a useful, but complicated, way to partition the feature space?

We have added a new main text figure (Figure 3A, reproduced below) to address these concerns, and discuss our findings in the first subsection of the Results section (reproduced below). In short, we find that CPT-1 outperforms several baselines that use unweighted averaging, especially in the high-sensitivity regime.

“To conclusively establish our claims about the value of supervising on DMS, we compared the full CPT-1 model with several additional baselines (Figure 3). First, we compared the performance of CPT-1 with alternatives that do not rely on the DMS data as much (Figure 3A). Specifically, we compared CPT-1 with unweighted averaging of EVE and ESM-1v, unweighted averaging of randomly selected features, and unweighted averaging of the features selected by our feature selection procedure. CPT-1 outperforms all of these alternatives, especially in the clinically relevant high-sensitivity regime. In particular, unweighted averaging of DMS-selected features performs worse than averaging ESM-1v and EVE, indicating that training on DMS goes beyond selecting features; the learned coefficients are essential to high performance.”

7

The authors pick 5 DMS datasets to train in. But, there are hundreds of available DMS datasets, many of which are focused on human disease-relevant. Some of these datasets outperform the ones the authors chose. They should better justify their choice of these five - just stating that they wanted to have more uniform data is insufficient. They should also explore what would happen if more datasets were included. They suggest that having datasets from a single lab/assay is important. Is that really so? Or, can lab-to-lab and assay-to-assay bias be dealt with?

We address this concern in new figures (Figure 3B and Supplemental Figure S2, both reproduced below). In Figure 3B, we show that, within our original five datasets, training on more data improves performance.

In Supplemental Figure S2, we explore the possibility of training on a larger set of DMS datasets pulled from a recent collection. We find that training within our five original proteins yields better results than a random selection of the broader, more heterogeneous set (Figure S2, top). This

8

effect is larger if we compare our five original datasets to only the thirteen other datasets (Figure S2, bottom). Moreover, using all the available data does not outperform only using the five original datasets.

The CPT model enters a crowded space of predictors seeking to inform human disease variant interpretation. The authors should do a better job in the introduction and discussion of contextualizing CPT in this crowded space. Is CPT really better than, say, REVEL, which many clinicians use? If so, why? If not, why is it useful?

We have added a new analysis, Figure 1D (reproduced below), which shows that CPT-1 outperforms REVEL on genes that REVEL did not train on. We discuss in the introduction that we believe CPT-1 has state-of-the-art accuracy and improved robustness over existing approaches.

“Furthermore, for genes not used to train REVEL, an ensemble method widely used by clinicians, we demonstrate that our model CPT-1 compares favorably with REVEL. We therefore expect that our predictions are accurate and more robust than what has previously been available, and we publicly release predictions for all possible missense variants in 90% of human genes.”

There have been a variety of previous efforts to use functional assay data to train predictors. The current work leverages more sophisticated ML methods and improved features, but the authors should cite and at least briefly discuss how their work relates to the previous work.

Thank you for pointing this out. We have cited and added a sentence about Envision and VESPA, two previous works that use functional assay data to train predictors. Envision does not achieve high performance on disease variant prediction, and the released version of VESPA does not outperform ESM.

“Previous work has trained on DMS data [26,27], but these works did not match the performance of models supervised on clinical data.”

The authors use the classifications "benign or deleterious." These may not be the best terms. In general, clinicians classify variants into one of five categories (benign, likely benign, VUS, likely pathogenic, or pathogenic). The title suggests that CPT predicts "disease variant effects." So, is it a pathogenicity predictor? If so, shouldn't the classes be benign and pathogenic? Or, if it is predicting variant effects rather than variant pathogenicity, which is what training on DMS data would suggest, then maybe terms like "functionally normal and functionally abnormal" would be better? Since one the authors led the development of the REVEL pathogenicity predictor, I think the authors can do a better job of clarifying whether CPT is a pathogenicity predictor and using appropriate terms.

10

We have updated our language throughout the paper to reflect that we train on “*functionally normal*” versus “*functionally abnormal*” in DMS data, and that this procedure transfers well to *pathogenicity* prediction. We have also more comprehensively shown that the same procedure improves functional assay prediction (Figure 1E).

They use Clinvar variants to validate the model. Clinvar is notoriously full of old and low-quality interpretations, which could actually make CPT appear worse than it is. It would be interesting to see how CPT performs on variants from at least 1 star submitters in the last 5-10 years.

Thank you for the suggestion. We have replaced our main evaluation dataset with 1 star ClinVar variants released since 2017. This new dataset improves the performance of CPT-1 as well as its margin over baselines.

The authors could do a much better job of labeling plot axes in figures. Many plots have labels like "Fraction" or "Spearman's rho" or "AUROC," but in different figures different fractions of things are being compared (e.g. DMS data or Clinvar data, etc). Improving the specificity of the labels would help reader comprehension.

Thank you for the helpful suggestion. We have made several changes to figure axes.

The authors make a version of CPT that uses imputed features. This is what enables them to go from the relatively small fraction of human proteins for which EVE predictions are available to 90% of human proteins. However, they don't present the performance of the model using these imputed features in the main text, describing results only vaguely and pointing to the supplement. I think the authors should put a version of supplementary figure 6 in the main text and substantially expand the section of the paper dealing with the imputation. They should fully

11

and fairly describe the effects of using imputed features. They should say how many genes have EVE-derived features and how many have imputed features. I didn't check, but they should also note whether EVE-derived or imputed features were used for each prediction available in the GitHub repository.

We have added Figure 6 and the corresponding discussion to the main text. This figure shows the performance of CTP-1 with imputed EVE predictions, in the same manner as our full model. We have added statements of how many genes use each model in our full release.

12

The authors should upload their predictions to Zenodo or some other external, versioned repository that does not allow data deletion. That way, CPT predictions will be available outside the author's GitHub repository.

We have uploaded the whole-proteome prediction to Zenodo
(<https://doi.org/10.5281/zenodo.7954657).>

Specific comments

Page 2 - The authors write "Functional assays via deep mutational scanning (DMS) experiments have been used to measure the effects of missense variants in non-human systems at higher throughput." This is confusing because many of the DMS experiments that have been done have been in cultured human cell lines. Clarifying or omitting "non-human systems" seems advisable.

Thank you for pointing this out. We have omitted “non-human systems”.

Page 2 - The authors write "However, the effects of missense mutations often involve aspects of protein function that are specific to humans or species similar to humans." This statement should have a citation. Moreover, I'm not sure I agree. And, the logic of the paragraph, which starts by asserting (correctly) that EVE and ESM-1v draw information from large MSAs composed mostly of non-human sequences and also that they perform quite well at predicting the effect of human variants on disease, contradicts the quoted paragraph. The authors shoudl clarify their argument.

We have clarified this argument, reproduced below. Our point is that, in functional assay prediction, it has been shown that EVE and ESM do not come close to the reproducibility of the assay data. Moreover, the models can be improved by integrating them with such system-specific data. We postulated that a similar strategy could work for human variant effect prediction.

“While this signal is powerful, it is not sufficient to fully explain how an amino acid substitution impacts protein function. This limitation has been shown by several recent studies in the context of functional assay prediction, which have found that the accuracy of sequence variation methods is far from the reproducibility of functional assays [21-23]. Moreover, sequence variation methods can be significantly improved by learning on functional data for a specific system of interest [21,22].

Analagous to these results in functional assay prediction, we postulated that protein sequence models such as EVE and ESM-1v could be combined with more human-specific sources of information to improve their performance on variant pathogenicity prediction.”

13

Page 2 - The authors state "...but such models can be inaccurate due to unknown biases in the collection of these data." This is a vague statement that should be clarified and cited.

We have clarified this statement and cited two papers that discuss the issue in the context of comparing variant effect predictors:

“However, such models can be affected by circularity and bias in the collection of these data, such as the use of other computational predictors to generate variant annotations [24-25].”

Page 3/Figure 2 - The authors show that CPT improves specificity at high sensitivity, which is great. It might help the reader appreciate how important these gains are if the authors used real numbers of variants as opposed to fractions/proportions.

We have added a sentence providing an example of how many false positives are eliminated by our model compared to EVE and ESM-1v in our dataset:

“Out of 13,815 benign variants in our dataset, this corresponds to nearly 1,800 fewer false positives compared to EVE and over 5,500 fewer false positives compared to ESM-1v when classifying 95\% of pathogenic variants correctly.”

Page 4 - The performance of the approach on predicting held-out DMS datasets is quite low.and not really better than previous approaches. Why? What is the relationship between being able to predict DMS data (moderate) and clinical variants (pretty good)? A discussion of these matters would be interesting.

We have tested CPT-1 on a significantly expanded set (eighteen) of functional assay datasets which includes the original five (Figure 1E, Table S3). We observe fairly consistent improved performance of CPT-1 over both ESM-1v and EVE. Each point above the dotted line is a dataset where CPT-1 outperforms the shown baseline.

14

Page 4 - The authors again make the argument that variants have human-specific effects that aren't well represented by MSAs drawn from diverse organisms. Again, I think this assertion needs more justification/citation/explanation.

We have more precisely stated our belief here, in line with the description in the introduction:

“Concretely, these models collect protein sequences from the set of all known proteins and employ redundancy filtering for sequences with high similarity. This approach models broadly recurring constraints well, such as structural constraints of a fold. However, we postulated that the models may be disregarding useful signal about sequence variation in species that are closer to humans.”

Page 4 and elsewhere - The authors use the phrase "local-timescale alignment." I think they mean an alignment of closely related vertebrates and mammals. If so, then I suggest they ditch or introduce the jargon which many will not understand. If they mean something else, they should explain.

We have removed this terminology.

15

Page 4 - The authors assert "These traits mean that conservation in these alignments is likely to be non-redundant with EVE and ESM-1v while being more specific to function and organism." They should examine the redundancy of conservation within their alignments as compared to those used for EVE/ESM-1v.

We have provided concrete numbers of what we mean by redundancy. For example, EVE downweights sequences to treat a cluster of 80% similar sequences as one observation. However, the average 100 vertebrate alignment contains 44 sequences that are within 80% sequence identity of the human protein, and our features treat each of these as one observation.

Page 4 - The authors state that others have previously shown that local-timescale alignments are powerful predictors of pathogenicity. Then, they go on to analyze how their own local timescale alignment is a powerful predictor of pathogenicity. It would be helpful for the reader to know what is new here, or if the authors are just reproducing what others have done/shown.

We have changed the language to precisely state that our main contribution is showing that this signal is non-redundant with much more powerful sequence variation methods.

Page 4- "Furthermore, if a mutant allele occurs at all in either the 100-vertebrate or 30-mammal MSA, it can be inferred to be benign with high probability (Figure 3B)" The authors could and should be more quantitative here.

We have expanded this description and provided numbers in the main text:

“Furthermore, if a variant occurs at all in either the 100-vertebrate or 30-mammal MSA, it can be inferred to be benign with high probability (Figure 4B). Concretely, mutant (more precisely, non-reference in human) alleles that appear at the same position in the reference genome for at least one other vertebrate are 82% benign, and those that appear for at least one other mammal are 94% benign. In contrast, variants that are not the reference allele in any other vertebrate are 61% pathogenic.”

Pages ^4^/_5_ - The authors note that their version of CPT with no vtMSA performs substantially worse on clinical data. It's interesting that it really craters at high sensitivity. Why is that? The authors should explore/explain.

We provide some example numbers in the main text that show that the vertebrate alignment features identify many variants that have ambiguous EVE scores as clearly benign:

“This margin can be partially explained in terms of the feature presented in Figure 4B regarding frequency of variants in the vertebrate alignments. Suppose we set both EVE and CPT-1 to predict variants with a sensitivity of 99%. If a variant is predicted as pathogenic by EVE but

16

appears as the reference allele for a non-human vertebrate, CPT-1 predicts it as pathogenic only 54% of the time. In contrast, if a variant is predicted as pathogenic by EVE and does not appear as the reference allele for a non-human vertebrate, CPT-1 predicts it as pathogenic 99% of the time.”

Page 5/Figure 3D - The authors highlight two genes where vtMSA is particularly important. Related to the previous comments, I feel this analysis could be improved significantly. Why were these genes chosen? What makes vtMSA useful for a gene (e.g. is it having a deeper MSA/gene length? Something else?)? Additionally, what variants does the vtMSA feature tend to get wrong?

We have removed the discussion of specific genes and instead discussed some general traits of the 10% of genes where vtMSAs help the most. We have also added text about where vertebrate MSAs would be wrong:

“At a per-gene level, adding vertebrate alignments is neutral or beneficial to the performance of CPT-1 in 84% of genes. The genes where vertebrate alignments help the most (top 10%) are more challenging genes in general, with lower average AUROCs for CPT-1, ESM-1v, and EVE. In addition, they have more shallow MSAs (average depth of 6,600 compared to 10,000 in all genes) and are longer (68% are longer than 1000 amino acids, compared to 44% in all genes). These discrepancies suggest that vertebrate alignments may be more useful in more complex human genes, which are more recently evolved and harder to model through general sequence homology.”

“CPT-1 could make an incorrect prediction for variants that are pathogenic in humans but appear as the reference allele in another vertebrate, but we find that very few such variants exist.”

Page 5 - The authors mention "a hand-designed feature that combines a known structure with conservation in the EVE MSA (Methods)." They should say more about it in the main text.

We have significantly expanded the description of this feature in both the main text and Methods. In the main text, we write:

“First, we included multiple versions of the deep neural network ProteinMPNN (which takes structure as input). Second, we included two hand-designed features that combine a known structure with conservation in the EVE MSA. For the latter features, we aimed to compute wild-type and mutant frequencies conditioned on the structural environment being the same as in the human protein. To achieve this, we first find for each position all other positions which are in contact in the AlphaFold structure. We then filter the EVE MSA to sequences where these positions have the same amino acids as in the human sequence. However, we perform this filtering using only a maximum of two contact residues, to ensure the number of sequences does not become too small. We define these features precisely in the Methods.”

17

Page 6 - Many readers of a generalist journal like GB will be unfamiliar with model perplexity. It should be introduced.

Upon re-examining this analysis, we found that the relationship between ESM perplexity and gene AUROC is highly confounded by the gene length effect that is described in a separate figure. In addition, MSA depth is no longer a significant predictor of EVE and ESM performances in our new dataset. We have therefore removed this discussion.

Page 6 - The authors "imputed all features that depend on the EVE MSA across genes." They should present an analysis of the quality of these imputed features.

We assess the quality of these features in Figure 6C-D, which compare full CPT-1, CPT-1 with imputed features, and CPT-1 with those same features removed. Imputation improves model performance but there is a gap to full CPT-1:

Page 6 - The authors state "We assessed this version of CPT-1 in the EVE dataset of

preprocessed ClinVar variants and found that the model still outperformed EVE and ESM-1v." Here, the authors make vague claims about the performance of the expanded CPT model, referring readers to the supplementary figure. Performance data should be presented in main figures.

These results are now presented in Figure 6A-B:

18

Page 6/supplementary figure 6 - The avg. per-gene AUROC for EVE predictions on "true" EVE genes is substantially better than the global AUROC. This seems weird to me, and should be explained.

We have added a brief discussion of this point. EVE is essentially a separate neural network for each gene. Therefore, variant scores are very well calibrated within each gene but it is difficult for the model to capture overall differences in mutation tolerance between genes.

19

**Second round of review**

**Reviewer 1**

The authors did a good job at addressing my concerns. They performed new experiments and made the code and data available. They also clarified the contribution of their method in terms of specificity gain. Although the method is not strikingly novel, there are new findings, e.g. the contribution of vertebrate alignments, that are clearly worth sharing with the community.

I still have one concern that I would like to be explicitly addressed in the final version of the manuscript. The authors mention some of the limitations of the work in the discussion, which is good. Nevertheless, I see the dependency on EVE scores as a major limitation for large scale applicability, given the amount of resources needed to train each EVE model on each query protein. This issue should be addressed in the main text, with some comments on possible solutions to overcome it. Based on the plot reported by the authors in their responses to the reviewers, it looks like there are much cheaper alternatives (e.g. GEMME) that could lead to the same performance.

**Reviewer 2**

The authors have dealt with my concerns adequately, and I have no further comments.

**Authors Response**

To address Reviewer 1's comment regarding GEMME and VESPA, we have added a paragraph in the Discussion section (highlighted in red) and also added a supplementary figure (Figure S6) in Additional File 1.
